# Supplementary material for: Beyond Diagnosis and Comorbidities—A Scoping Review of the Best Tools to Measure Complexity for Populations with Mental Illness
Source: Diagnostics (Basel). 2024 Jun 19;14(12):1300. doi: 10.3390/diagnostics14121300 (PMC11203348; doi:10.3390/diagnostics14121300)
Supplement: Supplementary file 1 [file diagnostics-14-01300-s001.zip › diagnostics-2966314-supplementary.pdf]

Supplemental information

PRISMA 2020 flow diagram for new systematic reviews which included searches of databases, registers and other sources

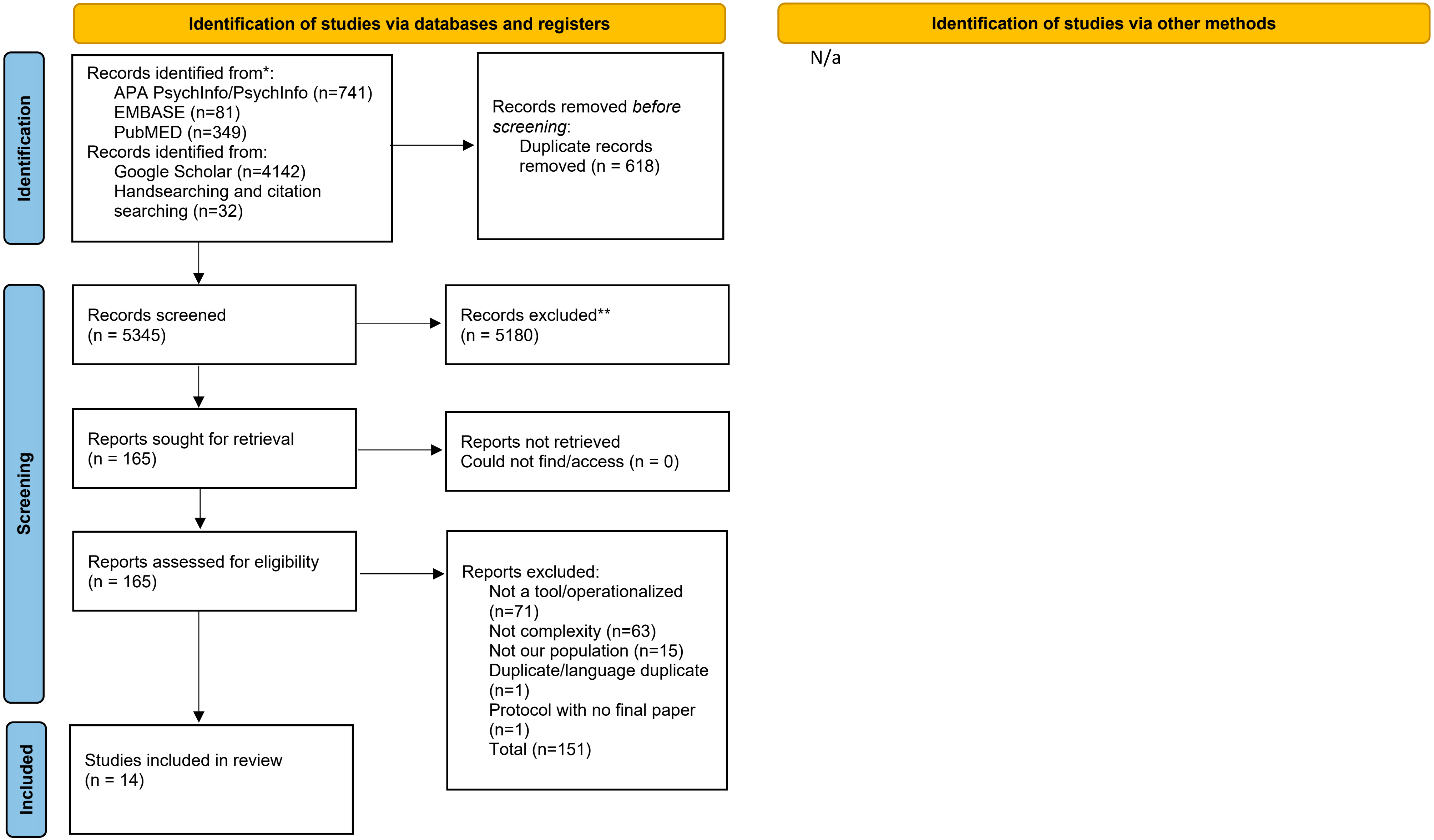

*Consider, if feasible to do so, reporting the number of records identified from each database or register searched (rather than the total number across all databases/registers).
**If automation tools were used, indicate how many records were excluded by a human and how many were excluded by automation tools.

From: Page MJ, McKenzie JE, Bossuyt PM, Boutron I, Hoffmann TC, Mulrow CD, et al. The PRISMA 2020 statement: an updated guideline for reporting systematic reviews. BMJ 2021;372:n71. doi: 10.1136/bmj.n71. For more information, visit: http://www.prisma-statement.org/
